# Exploring the correlation between serum fibroblast growth factor-21 levels and Sarcopenia: a systematic review and meta-analysis

**DOI:** 10.1186/s12891-023-06641-1

**Published:** 2023-06-29

**Authors:** Hao Liu, Xia He, Xiao-Yan Deng, Jing-Lu Yan

**Affiliations:** 1 School of Health Preservation and Rehabilitation, Chengdu University of Traditional Chinese Medicine, Chengdu, 610075 China; 2 Affiliated Sichuan Provincial Rehabilitation Hospital of the Chengdu University of Traditional Chinese Medicine, Chengdu, 611135 China; 3 Tianhui Town Community Health Center, Chengdu, 610081 China

**Keywords:** Sarcopenia; Fibroblast growth factor-21; Correlation

## Abstract

**Background:**

Fibroblast growth factor 21 (FGF-21) plays an important role in the growth and metabolism of skeletal muscle cells. This study aims to systemically review the evidence regarding the relationship between FGF-21 levels and Sarcopenia, as well as the related influential factors.

**Methods:**

This review was conducted according to the PRISMA guidelines. We comprehensively searched PubMed, EMBASE, the Web of Science, Scopus, and Chinese Databases (CNKI, Wan Fang, VIP, and CBM) up to 1 May 2023. 3 investigators performed independent literature screening and data extraction of the included literature, and two investigators performed an independent quality assessment of case-control studies using the Joanna Briggs Institute (JBI) tool. Data analysis was performed using Review Manager 5.4 software. For continuous various outcomes, mean difference (MD) or standard mean difference (SMD) with 95% confidence intervals (CIs) was applied for assessment by fixed-effect or random-effect model analysis. The heterogeneity test was performed by the Q-statistic and quantified using I^2^, and publication bias was evaluated using a funnel plot.

**Results:**

Five studies with a total of 625 cases were included in the review. Meta-analysis showed lower BMI in the sarcopenia group [MD= -2.88 (95% CI, -3. 49, -2.27); *P* < 0.00001; I^2^ = 0%], significantly reduced grip strength in the sarcopenia group compared to the non-sarcopenia group [MD = -7.32(95% CI, -10.42,-4.23); *P* < 0.00001; I^2^ = 93%]. No statistically significant differences in serum FGF21 levels were found when comparing the two groups of subjects [SMD = 0.31(95% CI, -0.42, 1.04); *P* = 0.41; I^2^ = 94%], and no strong correlation was found between the onset of sarcopenia and serum FGF21 levels.

**Conclusion:**

The diagnosis of sarcopenia is followed by a more significant decrease in muscle mass and strength, but there is a lack of strong evidence to support a direct relationship between elevated organismal FGF21 and sarcopenia, and it is not convincing to use FGF21 as a biological or diagnostic marker for sarcopenia. The currently used diagnostic criteria for sarcopenia and setting of cut-off values for each evaluation parameter no longer seem to match clinical practice.

## Introduction

Skeletal muscle is a multifunctional, multi-targeted tissue in the human organs. The maintenance of skeletal muscle morphology is influenced not only by the neurotrophic effects of the nerves that innervate its activity but also by several cytokines [1]. The European Society of Parenteral and Enteral Nutrition (ESPEN) defines sarcopenia as a syndrome characterized by progressive and generalized muscle loss [2], often occurring in a variety of systemic diseases, such as aging, malnutrition, inflammation, mitochondrial myopathy, and cancer cachexia [3, 4]. In particular, increased levels of inflammatory cytokines because of persistent inflammation are strongly associated with loss of skeletal muscle mass [5]. However, according to the European Working Group on Sarcopenia in the Elderly 2 (EWGSOP2) criteria, only a decrease in the strength, mass, and quantity of skeletal muscle can be included in the category of sarcopenia [6, 7]. This method only diagnoses sarcopenia from the behavioral perspective but does not touch the microscopic pathological changes of skeletal muscle cells, even though it has almost no reference value for the diagnosis of sarcopenic obesity [8]. The etiology and pathogenesis of sarcopenia are inherently complex and influenced by multiple factors [2], endocrine disorders, changes in glucose and lipid metabolism, and fasting are all involved in the pathological process of muscle atrophy [9]. During the pathological response to muscle atrophy, compensatory response deficiencies of the mitochondrial respiratory chain and impaired glucose (Glu) uptake and storage by skeletal muscle have both been identified by researchers [10]. To date, little is known about the pathological signaling pathway regulating atrophy associated with skeletal muscle myocyte senescence.

Fibroblast growth factor 21, which is one of three endocrine growth factors in the FGF superfamily. Not only does it exert its insulin-induced glucose uptake through FGF receptors and the cofactor β-klotho [11], but it is also a secreting myokine [12]. Up until now, the metabolism-related pathways by which FGF21 affects skeletal muscle mass are still in the hypothetical conjecture stage, but most investigators believe that serum FGF21 levels have a positive correlation with aging sarcopenia [13]. The expression of FGF21 is almost undetectable under healthy conditions [12] and is highest in centenarians. Due to the irreversible nature of aging, degeneration of different body tissues and changes in metabolic activity may affect the synthesis and release of FGF21. The animal-based studies and several substudies of clinical trials on sarcopenia that we have reviewed so far have found that muscle-dependent elevations of FGF21 may be associated with muscle aging, myocyte atrophy, and FGF 21 was once proposed as a specific serum candidate marker for sarcopenia [14-16]. For FGF21, which has a complex physiological effect and an extremely wide range of targets, there is no consensus on what effect it has on the muscular system, and it is not even clear whether this effect is a positive promoter or a negative aggressor.

Although our understanding of FGF21’s systemic effects has increased, its direct effects on muscle function are still not fully understood. The histological diagnostic technique continues to be the gold standard for sarcopenia diagnosis. The goal of this study is to identify and investigate the pathogenesis and pathological process of sarcopenia, as well as the intersection of serum FGF21 levels and the pathological process of sarcopenia.

## Materials and methods

The protocol of this systematic review was developed and submitted to PROSPERO, and the registration number is (CRD42022362885). This meta-analysis was conducted according to the Preferred Reporting Items for Systematic Reviews and Meta-Analyses (PRISMA) statement [17].

### Search strategies

A systematic search of the literature was conducted using electronic databases (PubMed, Embase, Scopus, Web of Science, and Chinese databases (CNKI, Wan Fang, VIP, and CBM)) from inception up to May 1, 2023. The following combination of search criteria was used: “fibroblast growth factor” and “sarcopenia,“ and included their synonyms and abbreviated forms.

### Eligibility criteria

Articles were included when they fulfilled the following criteria:

(i) Studies performed in clinically diagnosed Sarcopenia patients, based on the European Working Group on Sarcopenia in the Elderly 2 (EWGSOP2) or European Society of Parenteral and Enteral Nutrition (ESPEN) criteria;
(ii) The study should include serum FGF levels, extractable correlation coefficients, means, and standard deviations;
(iii) Original literature is all published literature.

### Exclusion Criteria

(i) Trials without designed control groups;
(ii) Studies with non-human subjects;
(iii) Outcome data that could not be extracted properly;
(iv) Reviews, case reports, conference abstracts, and studies for which full text was not available.

### Study selection

After the removal of duplicates, we individually assessed every study using the inclusion and exclusion criteria to determine its eligibility to be included in the systematic review and meta-analysis. Publications were first screened based on titles and abstracts, and afterward, the full text was examined. The reviewers screened the literature independently, and any disagreements that arose were resolved through discussion and negotiation between the two.

### Data extraction and management

The data and information extracted from each study included: study type, sample size, number of subjects, age, percentage of males and females, muscle condition, and serum FGF21 levels. We are most interested in the statistical analysis of correlations between serum FGF21 and skeletal muscle mass, grip strength, and body mass index in patients with sarcopenia. We rigorously extracted correlation indices and standardized transformations of statistical indicators of interest and of different types concerning relevant statistical methods to reduce the risk of bias when combining effect sizes.

### Assessment of study quality

Quality assessment of case-control studies by using the Critical Appraisal Tools from the Joanna Briggs Institute (JBI) Centre for Evidence-Based Health Care, Australia [18]. The tool contains 10 entries.

1. Were the groups comparable other than the presence of disease in cases or the absence of disease in controls?
2. Were cases and controls matched appropriately?
3. Were the same criteria used for the identification of cases and controls?
4. Was the exposure measured in a standard, valid, and reliable way?
5. Was exposure measured in the same way in cases and controls?
6. Were confounding factors identified?
7. Were strategies to deal with confounding factors stated?
8. Were outcomes assessed in a standard, valid and reliable way for cases and controls?
9. Was the exposure period of interest long enough to be meaningful?
10. Was the appropriate statistical analysis used?

The assessors made “yes,“ “no,“ “unclear,“ and “not applicable” judgments for each evaluation item. Two researchers alone assessed the included literature, and where there was disagreement, we discussed it to reach a consensus.

### Statistical analysis

Due to the relatively limited number of clinical studies exploring the correlation between human FGF21 levels and sarcopenia and the scattered observations indexes of interest selected for each study. Before meta-analysis, we first performed a descriptive analysis of the included studies and then summarized the same observation indexes for statistical pooling. Finally, we selected three groups of observations—body mass index, muscle strength, and skeletal muscle mass index—for statistical pooling analysis. We not only investigated the effect of different the three groups of observables on the FGF21 levels in sarcopenic patients but also, in turn, explored the pathological changes in human FGF21 levels and observables of interest before and after the diagnosis of sarcopenia.

Meta-analysis was performed using Review Manager (RevMan) Version 5.4 for Windows (Cochrane Collaboration, http://ims.cochrane.org/revman) to explore if some variables of the characteristics of all samples/studied or differences in some characteristics between those having sarcopenia and those without were significant moderators. To exclude bias due to differences in FGF21 measurement levels between studies, we preprocessed the data for parameter harmonization before meta-analysis. For continuous various outcomes, mean difference (MD) or standard mean difference (SMD) with 95% confidence intervals (CIs) was applied for assessment by fixed-effect or random-effect model analysis. Study heterogeneity was measured using the chi-squared and I-squared statistics, with chi-squared *p* ≤ 0.05 and I-squared ≥ 50% indicating the presence of significant heterogeneity. Publication bias was assessed with a visual inspection of funnel plots [19] for outcomes with at least 10 studies.

## Results

### Search results and inclusion

**Fig. 1:**
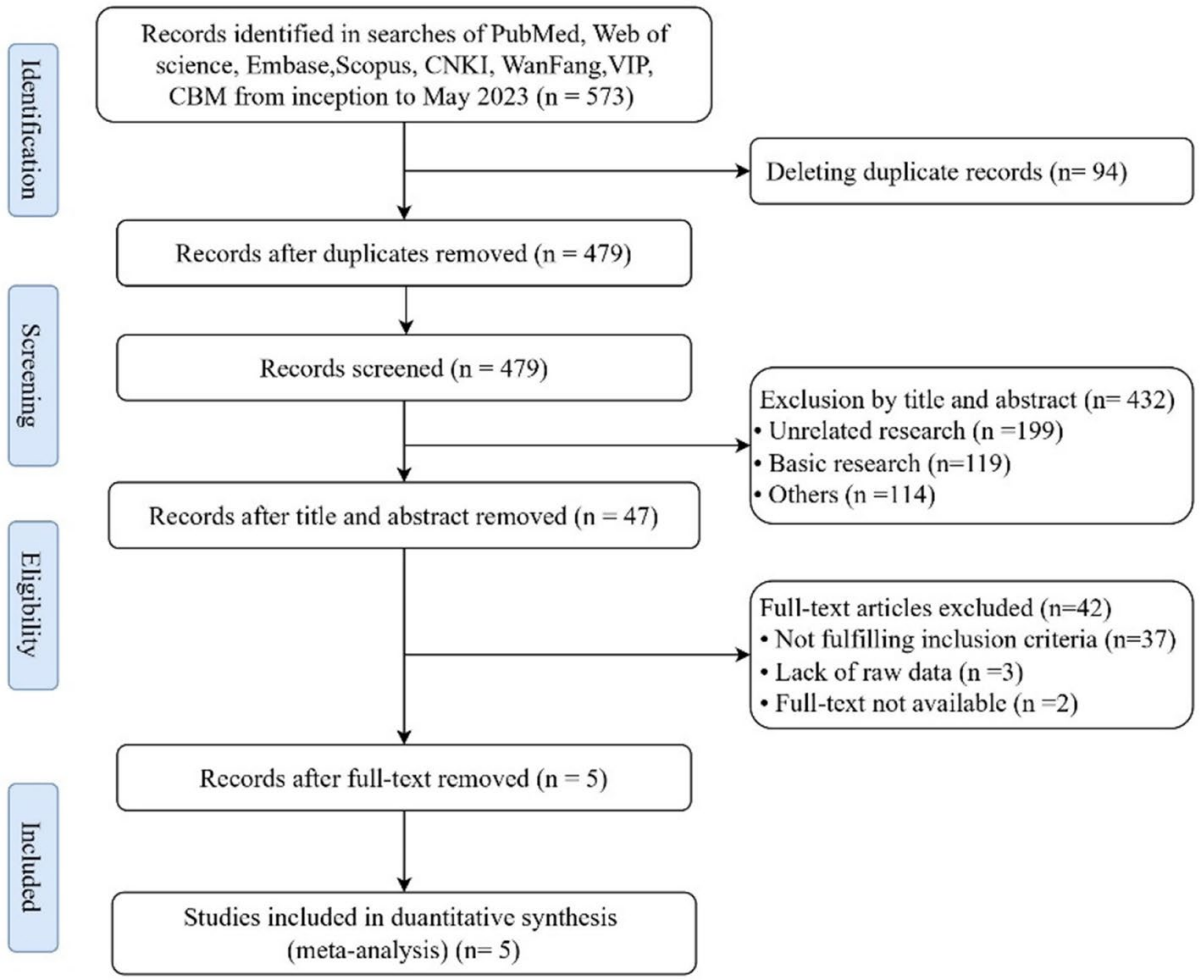
The inclusion process and final results

This study followed the PRISMA flow chart for literature screening. A total of 573 pieces of literature were initially retrieved. After the removal of 94 duplicates, 479 pieces of literature were screened based on titles and abstracts, and 47 pieces of literature were identified as potentially eligible. After checking the full text for detailed information and data extraction, 5 pieces of literature were included in this review [5, 20-24]. The summary of the screening process is presented in Fig. 1.

### Study and patient characteristics

Studies and patients’ characteristics are summarized in Table 1. The 5 meta-analyzed studies included a total of 625 participants (223 with sarcopenia and 402 without). The majority of the studies were conducted in Asia and published in the last five years. The majority of the subject populations in the included studies were from outpatient and community hospitals; in only one of the included studies was the subject group all female, and after excluding this study, the gender difference in subjects with sarcopenia was analyzed, with a ratio of almost 1:1 between males and females.

**Table 1:** Characterization of the studies included in the systematic review

Authors	Country	Design	Participants											
			Sarcopenia						Non-sarcopenia					
			Sample (n)	Age	BMI	FGF21	SMI	HG	Sample (n)	Age	BMI	FGF21	SMI	HG
Lu L (2017) [20]	China	Case control	78	64 ± 8	22.8 ± 3.3	74.1 ± 26.6	4.99 ± 0.30	16.5 ± 3.3	172	62 ± 8	25.5 ± 3.1	72 ± 30.9	6.17 ± 0.61	20.1 ± 3.6
Li C W 2019 [6]	China	Case control	56	72 ± 6.54	21.4 ± 3.09	49.9 ± 10.9	5.7 ± 0.78	23.2 ± 7.34	56	65.2 ± 4.0	24.6 ± 3.02	58 ± 13.9	7.04 ± 0.85	29.5 ± 8.93
Bag S R 2021 [23]	Turkey	Case control	43	80.3 ± 2.4	30.7 ± 5.4	82.4 ± 28.5	6.9 ± 0.44	16.4 ± 2.14	45	76.3 ± 2.0	29.2 ± 3.6	54.1 ± 17.7	7.4 ± 0.6	25.2 ± 3.48
Jung H W 2021 [21]	Korea	Case control	21	71.9 ± 4.7	24.0 ± 3.3	71.6 ± 45.3	5.4 ± 0.78	21.9 ± 6.2	104	68.6 ± 6.5	26.5 ± 3.1	29.5 ± 26.7	7.03 ± 1.06	28.9 ± 9.3
Ofazoglu U 2022 [24]	Turkey	Case control	25	57.5 ± 14.1	25.2 ± 4.1	55.8 ± 22.4	8.9 ± 1.1	23.1 ± 3.9	25	58.8 ± 10	28.8 ± 5.4	175.3 ± 382.9	13.1 ± 1.7	34.4 ± 6.5

*BMI *Body mass index, *FGF21 *Fibroblast growth factor-21, *SMI *Skeletal muscle index, *HG *Handgrip strength

The original studies all recorded the biological indicators of the subjects, and we mainly extracted the body mass index (BMI), Serum fibroblast growth factor 21 levels, Skeletal muscle index (SMI), and grip strength. Four of the studies used SMI for the evaluation of muscle mass, and only one used Relative skeletal muscle index (RSMI). Reviewing the original study regarding RSMI calculation formulas, its principle meaning is not different from SMI.

### The results of the quality assessment

According to the JBI critical appraisal tools, all five case-control studies were of high quality for entries 1–5, with two not controlling for confounding factors. The quality assessment details are summarized in Table 2.

**Table 2:** Results of the quality assessment of included studies

Author (Year)	Were the groups comparable other than the presence of disease in cases or the absence of disease in controls?	Were cases and controls matched appropriately?	Were the same criteria used for identification of cases and controls?	Was exposure measured in a standard, valid and reliable way?	Was exposure measured in the same way for cases and controls?	Were confounding factors identified?	Were strategies to deal with confounding factors stated?	Were outcomes assessed in a standard, valid and reliable way for cases and controls?	Was the exposure period of interest long enough to be meaningful?	Was appropriate statistical analysis used?
Lu L (2017) [20]	√	√	√	√	√	×	×	√	√	√
Li C W 2019 [6]	√	√	√	√	√	×	/	√	√	√
Bag S R 2021 [23]	√	√	√	√	√	√	√	√	√	√
Jung H W 2021 [21]	√	√	√	√	√	√	√	√	√	√
Ofazoglu U 2022 [24]	√	√	√	√	√	×	×	√	/	√

√= Yes; ×= No; /=Unclear; -=Not applicable

### Meta-analysis

#### Changes in BMI after diagnosis of sarcopenia

**Fig. 2:**
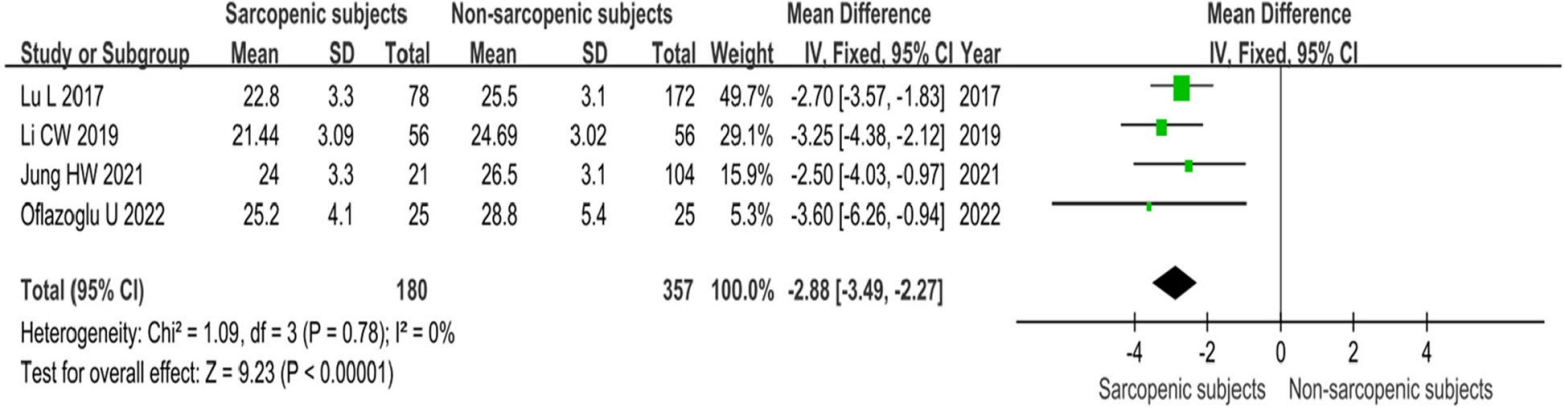
Meta-analysis of differences between groups in BMI in sarcopenia

The combined findings of five studies revealed a significant difference in BMI between subjects with and without sarcopenia. Patients with sarcopenia have a lower BMI than those without it [MD=-2.84 (95% CI, -3. 06, -1.89); *P* < 0.00001; I^2^ = 79%]. However, heterogeneity between studies exceeded 50% sensitivity analysis revealed Heterogeneity originated from Soytas’ study. After reviewing the article, we found that the subjects included in this study were older (mean age: 80.3 (78.6–81.9) and had a closer BMI between the two groups. Heterogeneity decreased to 0 after excluding this study, and the results of the meta-analysis were more reliable [MD= -2.88 (95% CI, -3. 49, -2.27); *P* < 0.00001; I^2^ = 0%] (Fig. 2).

#### Correlation between FGF21 and Sarcopenia

**Fig. 3:**
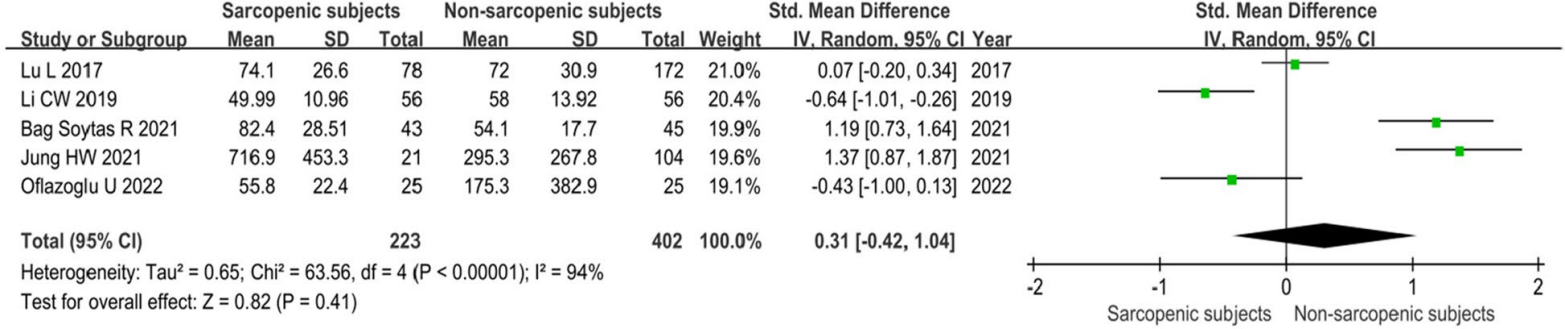
Meta-analysis of differences between groups in FGF21 in sarcopenia

The levels of serum FGF21 in sarcopenia and non-sarcopenia were combined and it is easy to find from the results that there was a large heterogeneity between the studies and even conflicting conclusions were drawn. Statistical findings were more consistent with the results of the descriptive analysis, with no significant difference in FGF21 levels between patients with and without sarcopenia [SMD = 0.31(95% CI, -0.42, 1.04); *P* = 0.41; I^2^ = 94%] (Fig. 3). We have preprocessed the data as well as controlled for confounding factors that may produce higher heterogeneity before combining the analyses; this, however, did not affect the final statistical results.

#### Decreased grip strength after diagnosis of sarcopenia

**Fig. 4:**
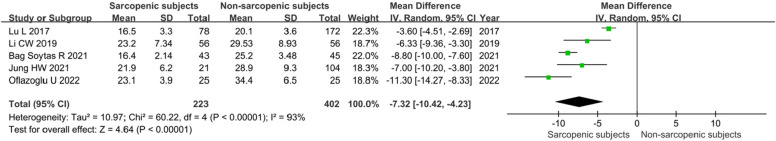
Meta-analysis of differences between groups in grip strength in sarcopenia

Grip strength was summarized with data from five studies. The results of the analysis indicate that the decrease in grip strength level after being diagnosed with sarcopenia is extremely significant [MD = -7.32; (95% CI, -10.42 ,-4.23); *P* < 0.00001; I^2^ = 93%] (Fig. 4), which is the most direct and significant clinical manifestation of sarcopenia. Subjects included in the study spanned an age range of more than 30 years, and grip strength decreased with increasing age, the is perhaps the main source of the greater heterogeneity.

#### Changes in skeletal muscle mass index (SMI) after diagnosis of sarcopenia

**Fig. 5:**
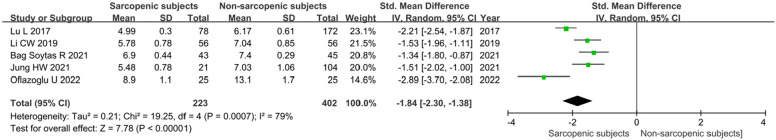
Meta-analysis of differences between groups in skeletal muscle mass index in sarcopenia

The means to evaluate muscle mass in sarcopenia patients varied between studies, and four used SMI to evaluate skeletal muscle mass while only one used the Relative skeletal muscle index (RSMI). Therefore, a meta-analysis was performed using the SMD model, and the results of the combined analysis of the data showed that patients with sarcopenia had a significantly lower SMI than controls [SMD= -1.84(95% CI, -2.30, -1.38); *P* < 0.00001; I^2^ = 79%] (Fig. 5). Furthermore, In the descriptive analysis, patients with sarcopenia showed more severe complaints in the questionnaire administered to the effect of decreased skeletal muscle mass and muscle loss on the ability to perform daily living.

### Publication bias

As fewer original studies were included, in principle more than 9 original studies were included for publication bias to be meaningful.

## Discussion

The Asian Working Group for Sarcopenia (AWGS) 2014 consensus defined sarcopenia as “age-related loss of muscle mass, plus low muscle strength, and/or low physical performance” and specified cutoffs for each diagnostic component [25]. Clinical and research interest in sarcopenia has burgeoned internationally. AWGS updated diagnostic algorithms, protocols, and some criteria for “sarcopenia” in 2019 [26]. The diagnosis of sarcopenia is currently based on several assessment parameters, each of which lacks uniform criteria [14, 27]. Thus, it is possible that finding a biomarker may help in the clinical evaluation, diagnosis, treatment, and prevention of sarcopenia.

Recent studies reported that skeletal muscle is not only a producer but also a target of FGF21 because it expresses the β-klotho coreceptor and the Fibroblast Growth Factor Receptor (FGFRs) [11]. Due to the limited proliferative capacity of human myoblasts, the regulation of their size is determined by the coordinated balance between protein synthesis and protein degradation of muscle fibers. In Oost’s animal experiments investigating the control of mitophagy and muscle mass by FGF21, the muscle protein synthesis rate in FGF21 knockout mice was not significantly different from controls; instead, those mice that were fasted for 48 h had a nearly 70% decrease in muscle protein synthesis rates [28]. It follows that FGF21 does not affect protein synthesis in muscle, but we do not know whether elevated serum FGF21 accelerates protein degradation.

**Fig. 6:**
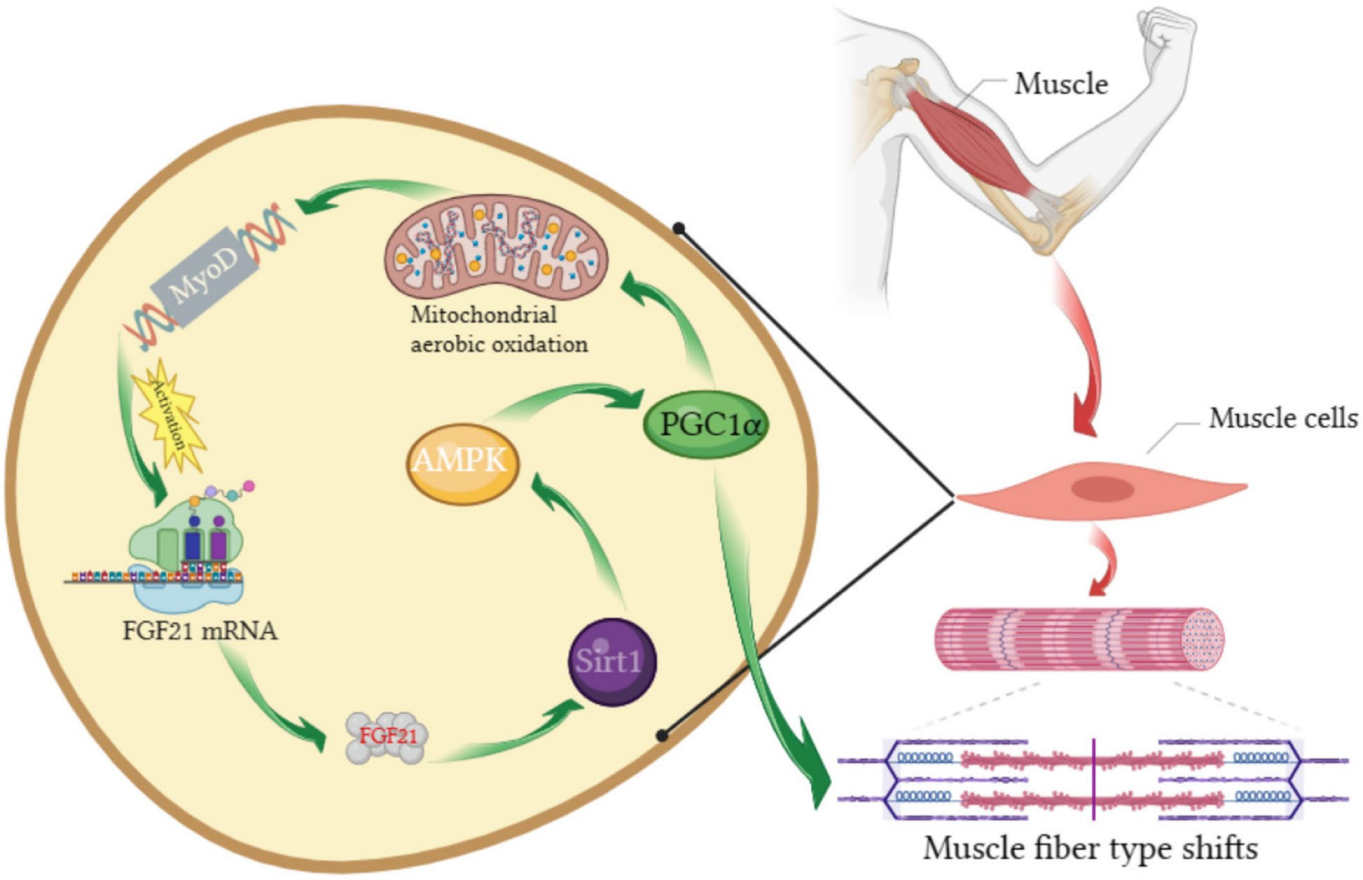
Muscle fiber type conversion by FGF21-SIRT1-AMPK-PGC1α axis

In humans, however, an elevated serum FGF21 is also the best predictor of risk for the development of several chronic diseases. Prolonged elevation of muscle-derived circulating FGF21 can lead to systemic inflammation, progeria syndrome, and premature death. The known molecular mechanisms underlying the pathology of sarcopenia, a condition associated with aging, include anabolic resistance and chronic inflammation [29]. With advances in metabolomics and molecular diagnostic techniques, research has uncovered a multi-effect link between more profound muscle dysfunction and disruptions in physio-logical homeostasis at the whole body level. Several studies have described in detail the multiple effects of FGF21 on muscle tissue, it should also be noted that muscle atrophy is always accompanied by muscle fiber type shifts, but the mechanism by which FGF21 regulates muscle fiber type shifts remains unclear. XY Liu studies [30] confirm that FGF21 activates the expression of early-stage myogenic genes and promotes myogenic differentiation through the FGF21-SIRT1-AMPK-PGC1α axis driven by muscle regulatory proteins and transcription factors of the MyoD family in a coordinated manner, finally resulting in the transition of the fiber type from larger anaerobic fibers to smaller aerobic fibers (Fig. 6) [31], leading to muscle mass loss. These data greatly extend the findings of our previous studies and provide a theoretical basis for new therapies to treat muscle diseases.

The incidence of sarcopenia increases with age, but it is likely to be reversible. BMI is an internationally used, simple, and convenient measure of body weight and health, and our statistical results show that there is a significant decrease in BMI in the short term after the diagnosis of sarcopenia. Summarizing the correlation between BMI and muscle mass across studies, the two showed a negative correlation. It seems that a higher BMI has a protective effect on episodic sarcopenia and its reversibility [32]. We also considered BMI as a possible confounder to control the possible effects of obesity on the association between serum FGF21 and sarcopenia. The apparent contradiction between the elevated circulating FGF21 in obese patients in a previous study [33] and the finding of sarcopenia in the present study may be an interesting mechanistic link between chronic metabolic conditions and muscle loss. However, these mechanistic aspects cannot be evaluated in the present study design and remain speculative [34]. The phenomenon of higher serum FGF21 levels in obese patients may be unlikely to influence the results of our study.

At this stage, our concern is that BMI does not adequately evaluate the composition and distribution of human tissues and does not distinguish the distribution of adipose tissue. Combined with the results of our study, we found that although the BMI of sarcopenic patients is significantly lower than normal levels, the presence of human adipose tissue is ignored. For those who are excessively obese and suffer from sarcopenia (defined as sarcopenic obesity), BMI, whether it measures body fatness or evaluates muscle status, is only a geometric description of the overall body tissue, neither of which can be used as a methodology to identify sarcopenia. The L.K. Chen [25] and I.Y.Jang [35] study adjusted ASM (Appendicular Skeletal Muscle Mass) by dividing by height^2^ for low muscle mass to capture the construct of sarcopenia associated with geriatric outcomes of fall, functional decline, and mortality, rather than adjusting ASM by dividing by weight or BMI, which better reflects the metabolic condition and sarcopenic obesity. ASM/BMI and FM/FFM (Fat Mass Index/Fat-Free Mass Index) may be the best evaluation indicators to respond to the muscle status of elderly patients in the pre-myasthenic phase [36, 37]. We strongly recommend a secondary analysis of completed or ongoing follow-up studies to explore the basis for geometric assessments that can identify sarcopenia.

Human muscle mass and strength decrease progressively with age, and it is estimated that healthy individuals lose approximately 1% per year after age 30 [38]. Once diagnosed with sarcopenia, the rate of muscle tissue aging accelerates dramatically. The results of our statistics are consistent with this. The earliest definition of a grip strength cut-off for sarcopenia was proposed by EWGSOP in 2010, and it has become the most commonly used grip strength diagnostic criterion for sarcopenia worldwide (20 kg for women and 30 kg for men) [7]. However, over the past decade, this criterion seems to be suffering from challenges, with research teams finding that it does not seem to accurately evaluate muscle strength changes in patients with sarcopenia. One of the primary reasons for this is that the cutoff was developed for European and North American populations, but the criterion has been widely adopted by populations outside of Europe and North America.

The reasons for the high heterogeneity of the results in our analysis are complex and varied. There was a lack of standardization in the method of grip strength measurement (including postural selection, body position placement, etc.) and the choice of the measurement site. Of course, it is not possible to generalize this as age increases and the age at which sarcopenia is diagnosed varies. Since fewer original studies were included, a rough subgroup analysis in terms of age can yet reveal a negative exponential relationship between age and grip strength, with this relationship becoming more significant with higher age. The more severe and persistent decline in instrumental activities of daily living brings about a progressive loss of independence in patients’ lives [39].

We summarized and analyzed the measures of muscle grip strength in the included studies, and the measurement sites chosen varied widely so that many clinicians have reported that the currently used grip strength criteria no longer meet the needs of clinical diagnosis and treatment. The Moreira VG study investigated the decline of grip strength with age in populations from around the world, and significant differences were found across ethnic groups [40]. Second, the various instruments used to identify grip strength loss may produce different measurement errors [41]. Therefore, the reported prevalence of “sarcopenia” may not be accurate [42]. In the absence of a simple “gold standard” for diagnosis, defining sarcopenia as grip strength below a cut-off value is not rigorous [43]. In addition, a persistent decline in the ability to live independently is increasingly mentioned at the first visit [39], and in the ensuing follow-up records, these populations complain of suffering more negative effects of incapacity (Falls, bone loss, etc. are more frequently recorded) [44].

Several animal studies have shown that skeletal muscle is the primary source of FGF-21 in the circulatory system [14, 15]. A recent study has shown that chronic elevation of circulating muscle-derived FGF-21 leads to systemic inflammation, premature aging, and premature death [45]. In addition, it has been concluded that serum FGF-21 levels have a positive correlation with primary sarcopenia [14, 28]. However, our combined statistics of published studies showed that the correlation between FGF21 and sarcopenia was diametrically opposed in different studies and that this correlation existed at a weak level, regardless of whether the relationship was positive or negative. Most surprising, however, was the absence of hypertrophy of skeletal muscle cells and functional recovery in skeletal muscle after treatment related to elevating serum FGF-21 levels was given to the patients [46]. In another study, it was found that strenuous exercise could reduce serum levels of FGF-21 and that muscle mass and strength were restored to some extent, but this was mostly identified as a reinforcing effect due to limb movement [47]. Whether FGF21 is a potential biomarker for the diagnosis of sarcopenia has not yet been confirmed by the strong results of studies.

### Limitations

Despite the preliminary understanding of FGF21 expression in myocyte effects, we still define sarcopenia by behavioral methods of diagnosis and evaluation, which makes the inclusion criteria of our study more lenient. Whether exploring the metabolic effects of FGF21 on myocytes from a microfoundational perspective or analyzing changes in muscle mass and strength from a behavioral perspective, the statistical conclusions we draw will be subject to a greater risk of bias from “Were cases and controls matched appropriately? Were the same criteria used for the identification of cases and controls?“.

## Conclusion

In conclusion, we analyzed the correlation between sarcopenia and FGF21 based on the paucity of original studies, and there is no strong evidence yet for a direct relationship between FGF21 and sarcopenia. The diagnostic criteria currently in use and the setting of cut-off values for each evaluation parameter no longer seem to meet the needs of clinical diagnosis and treatment. Sarcopenia is influenced by genetic and lifestyle factors that occur throughout the life course. More far-reaching inquiry is needed regarding the translation of pathophysiological knowledge into clinical diagnosis and service for therapeutic management, especially with the search for targeted biomarkers, nutritional interventions, and drug development as the focus of scientific exploration.

## Data Availability

The datasets generated and/or analysed during the current study are not publicly available, but are available from the corresponding author on reasonable request.
